# Sampling efficiency and nucleic acid stability during long-term sampling with different bioaerosol samplers

**DOI:** 10.1007/s10661-024-12735-7

**Published:** 2024-05-25

**Authors:** Kari Oline Bøifot, Gunnar Skogan, Marius Dybwad

**Affiliations:** 1 Norwegian Defence Research Establishment, P.O. Box 25, NO-2027 Kjeller, Norway; 2 Department of Analytical, Environmental and Forensic Sciences, King’s College London, 150 Stamford Street, London, SE1 9NH UK

**Keywords:** Air sampler, Bioaerosol, Sampling efficiency, Nucleic acid stability

## Abstract

Aerosol microbiome studies have received increased attention as technological advancements have made it possible to dive deeper into the microbial diversity. To enhance biomass collection for metagenomic sequencing, long-term sampling is a common strategy. While the impact of prolonged sampling times on microorganisms’ culturability and viability is well-established, its effect on nucleic acid stability remains less understood but is essential to ensure representative sample collection. This study evaluated four air samplers (SKC BioSampler, SASS3100, Coriolis μ, BioSpot-VIVAS 300-P) against a reference sampler (isopore membrane filters) to identify nucleic acid stability during long-term sampling. Physical sampling efficiencies determined with a fluorescent tracer for three particle sizes (0.8, 1, and 3 μm), revealed high efficiencies (> 80% relative to reference) for BioSampler, SASS3100, and BioSpot-VIVAS for all particle sizes, and for Coriolis with 3 μm particles. Coriolis exhibited lower efficiency for 0.8 μm (7%) and 1 μm (50%) particles. During 2-h sampling with MS2 and *Pantoea agglomerans*, liquid-based collection with Coriolis and BioSampler showed a decrease in nucleic acid yields for all test conditions. BioSpot-VIVAS displayed reduced sampling efficiency for *P. agglomerans* compared to MS2 and the other air samplers, while filter-based collection with SASS3100 and isopore membrane filters, showed indications of DNA degradation for 1 μm particles of *P. agglomerans* after long-term sampling. These findings show that long-term air sampling affects nucleic acid stability in both liquid- and filter-based collection methods. These results highlight bias produced by bioaerosol collection and should be considered when selecting an air sampler and interpreting aerosol microbiome data.

**Supplementary Information:**

The online version contains supplementary material available at 10.1007/s10661-024-12735-7.

## Introduction

In bioaerosol research, active air sampling is the most common method used. There are several different collection principles (e.g., impaction, impingement, filtration, and condensation growth), each with its advantages and disadvantages (Bhardwaj et al., 2021; Haig et al., 2016). Bioaerosol research has traditionally relied on culture-dependent methods, but in later years, there has been a shift toward molecular and sequence-based studies of airborne microorganisms (Hou et al., 2023; Mbareche et al., 2017). Molecular methods have the potential to greatly improve our understanding and identification of airborne microorganisms, as a large proportion of airborne microorganisms will not grow under standard laboratory conditions. It has also been reported that several factors, including environmental conditions and particle size, affect the culturability of microorganisms (Dybwad & Skogan, 2017; Lighthart & Shaffer, 1997; Peccia & Hernandez, 2006; Šantl-Temkiv et al., 2020). The shift from culture-dependent to culture-independent methods also changes the requirements to the air sampling strategy. For culture-based work, it is important to use a gentle air sampling technique to preserve culturability, especially for more stress-sensitive organisms such as gram negatives which are more prone to sampling stress than spores and gram positives (Zhen et al., 2013). Two commonly used collection principles for studying culturable airborne bacteria are impaction directly onto an agar plate at a low flow rate or gentle collection into a liquid buffer. On the contrary, for culture-independent methods, the biological state of the microorganisms is less important as long as the nucleic acids remain intact and can be recovered from the sample (Lindsley et al., 2017).

The air is generally regarded as a low biomass environment, outdoor air in particular. When investigating the aerosol microbiome and microbial diversity using shotgun sequencing there is a need for higher biomass yields than for amplicon-based methods (e.g., 16S rRNA gene amplicon sequencing). Not achieving high enough yields has typically been solved by pooling samples and/or performing whole genome amplification (Abd Aziz et al., 2018; Be et al., 2015; Yooseph et al., 2013). The use of high-volume air samplers (e.g., SASS3100 and Coriolis μ) and/or increased sampling time has proven to be an effective strategy to collect enough biomass for sequencing studies, and a typical prerequisite to collect enough biomass for shotgun sequencing of air samples (Cao et al., 2014; Gusareva et al., 2020; Hou et al., 2023; Leung et al., 2021). However, high flow rates and long sampling times increase the risk of negative side effects, such as desiccation, osmotic shock, and evaporation of sampling buffer. The stress factors induced by air sampling can impact culturability and viability, and in the worst case, lead to cell rupture and release/loss of nucleic acids (King et al., 2020; Zhen et al., 2013). Desiccation is a typical drawback for filter sampling (dry sampling), as collected material is surrounded by a continuous airflow which desiccates the cells (Bhardwaj et al., 2021). To process filter samples, particulate matter is extracted into a liquid, and in this process, nucleic acids can be released into the filter extract if cells are damaged. The nucleic acids may remain intact and be recovered for molecular analysis, but it is then important to process the whole sample volume to not lose free-nucleic acids and by that microbial diversity (Bøifot et al., 2020a, 2020b; Zhen et al., 2013).

To avoid desiccation of microbial cells during air sampling and maintain culturability and infectivity, collection into a liquid buffer (e.g., impingement and wetted-wall cyclone) is a common strategy (Lindsley et al., 2017). Although microbial cells and viruses might be protected from desiccation, evaporation of sampling buffer is a common problem for, e.g., impingement (SKC BioSampler) and wetted-wall cyclones (Coriolis μ). Evaporation can reduce the sampling buffer volume below the optimal volume for particle collection, thereby reducing collection efficiency, and it has also been shown that collected material can be reaerosolized and/or suffer from internal loss (Han & Mainelis, 2012; Lin et al., 1997; Riemenschneider et al., 2010; Rufino de Sousa et al., 2020). To compensate for evaporation, some liquid air samplers, such as the Coriolis μ and SASS2300, can replenish the sampling buffer during collection. However, this increases the air sampler’s complexity (design and operation) and the contamination risk due to inadequate and/or difficult cleaning of the fluidic system. In real-world environments, it will also be difficult to adjust the refill rate as changing temperatures and humidity will affect the evaporation rate, and contamination is a big concern in microbiome studies because of the sensitivity of shotgun sequencing (Eisenhofer et al., 2019). A recently commercialized alternative based on condensation growth technology, BioSpot-VIVAS, has also the advantage of avoiding desiccation of the cells. Additionally, it is considered to be a gentle and efficient collection principle, suitable for culturing and molecular methods, with a high concentration factor and free selection of collection buffer, convenient for downstream sample processing. However, the flow rate is low (8 L/min) compared to the high-volume air samplers (≥ 300 L/min) that have successfully been used in shotgun sequencing studies (Archer et al., 2019; Gusareva et al., 2019; Leung et al., 2021; Qin et al., 2020). Condensation growth has been evaluated in several chamber studies and has shown good recovery and preservation of various microorganisms (Degois et al., 2021; Nieto-Caballero et al., 2019; Pan et al., 2018; Raynor et al., 2021). During the recent pandemic, several studies have reported the successful use of condensation growth sampling to study the abundance and infectivity of SARS-CoV-2 (Banholzer et al., 2023; Fortin et al., 2023; Vass et al., 2022). However, there have been few studies comparing condensation growth with other collection principles using bacteria and molecular methods (Nieto-Caballero, 2021).

Many of the challenges faced by the different collection principles will become more prevalent with increased sampling time. This can cause microbial cells and nucleic acids to be differentially damaged due to varying degrees of resistance to sampling-associated stress factors, or the microorganisms can be lost through reaerosolization (Lemieux et al., 2019; Zhen et al., 2013). There are several considerations to be made when selecting an air sampler depending on study design and aim, e.g., sampling efficiency, compatibility with downstream processes, battery operation, low noise level, low weight (mobility), and high flow rate for high resolution or collection of sufficient biomass. The many factors that must be considered when selecting an air sampler are likely one of the reasons why there is a lack of standardized and harmonized methods within bioaerosol research. For decades, it has been highlighted that there is a need to standardize and harmonize methods to allow for comparison between studies to advance the field as different collection methods can yield different results (Cox et al., 2020; Griffiths & DeCosemo, 1994; Lemieux et al., 2019; Mainelis, 2020; Millner, 2009). To improve our understanding of different collection principles, several studies have sought to compare different collection principles and air samplers and how they can affect infectivity (Degois et al., 2021; Raynor et al., 2021), culturability (Dybwad et al., 2014), microbial diversity in real-world environments (Hoisington et al., 2014; Lemieux et al., 2019; Mbareche et al., 2018), cell damage (Zhen et al., 2013), RNA stability with low volume air samplers (Degois et al., 2021; Ratnesar-Shumate et al., 2021), DNA intactness (King & McFarland, 2012), and DNA and RNA stability (Guo et al., 2024; Zhen et al., 2018). Comparison of different air samplers in real-world environments has shown distinct differences in microbial diversity. Though the underlying mechanism for the differences is not well established, collection efficiency, reaerosolization, and degradation of nucleic acids are potential factors (Hoisington et al., 2014; Lemieux et al., 2019; Mbareche et al., 2018). Previous studies investigating DNA and RNA stability do not give a clear conclusion. Guo et al. (2024) showed that liquid sampling in general had a higher nucleic acid recovery than filter sampling, while Zhen et al. (2018) showed the opposite with spike experiments. Ratnesar-Shumate et al. (2021) found that there are no large differences between filter and liquid sampling, while Degois et al. (2021) found variability depending on virus species. Air sampler comparison studies, using aerosolized microorganisms to investigate effects of long-term sampling have only used viruses, and without high-volume air samplers commonly used in microbiome studies (Coriolis μ and SASS3100). In the new era of microbiome studies, there is an increasing need to ensure that representative samples are collected and maintained. Knowledge of nucleic acid stability during long-term sampling with different collection principles is therefore important as long-term sampling is a common strategy to collect enough biomass for metagenomic sequencing.

In this study, we investigated physical sampling efficiency and nucleic acid stability in an aerosol test chamber (ATC) for different collection principles during long-term sampling using Uranine and two model organisms, the gram-negative vegetative bacteria *Pantoea agglomerans* (PA) and the bacteriophage MS2 (MS2). Isopore membrane filters (reference) were compared towards four bioaerosol samplers, the high-volume air samplers SASS3100 (electret filter) and Coriolis μ (wetted-wall cyclone) commonly used in microbiome studies, BioSpot-VIVAS-300P (condensation growth) which has shown promising results in virus studies, and the well-established SKC BioSampler. We characterized their physical sampling efficiency for three different particle sizes (0.8, 1, and 3 μm) relative to a reference sampler using a fluorescent tracer (Uranine). We also investigated nucleic acid recovery and stability of PA (dsDNA) and MS2 (ssRNA) during short- and long-term sampling (10 min and 2 h, respectively) at two particle sizes (1 and 3 μm). We hypothesized that we would find a decrease in nucleic acid yields after long-term sampling. The results from this study could help interpret the suitability of different air samplers and collection principles for use in studies where long-term sampling is needed to obtain sufficient biomass (e.g., for shotgun sequencing).

## Materials and methods

### Evaluated air samplers

Five different bioaerosol samplers were included in this study (Table 1), BioSpot-VIVAS 300-P (hereafter referred to as VIVAS; Aerosol Devices Inc., Fort Collins, CO, USA), SASS3100 (hereafter referred to as SASS; Research International, Monroe, WA, USA), Coriolis μ with long time monitoring option (hereafter referred to as Coriolis; Bertin Technologies, Montigny-le-Bretonneux, France), SKC BioSampler (20 ml; hereafter referred to as BioSampler; SKC Inc., Eight Four, PA, USA), and isopore membrane filters (HTTP03700; hereafter referred to as isopore filters; Merck KGaA, Darmstadt, Germany). All air samplers were used according to the manufacturers’ instructions, except BioSampler which used a longer sampling time than recommended without the addition of mineral oil or glycerol.

**Table 1:** Air samplers used in this study. Information regarding sampling time and efficiency is given by the manufacturer. The face velocity was calculated for the isopore membrane filter and SASS3100 with the flow rates used in this study

Air sampler	Collection principle	Flow rate in study (L/min)	Recommended sampling time	Efficiency at 0.5 μm	Face/impaction velocity (m/s)
BioSampler 20 ml (225–9595)	Impingement (wet)	12.5	30 min–8 h	90%	313 (Lin et al., 2000)
BioSpot-VIVAS 300-P	Condensation growth tube (wet)	8	1 min–23 h and 59 min	> 90%	0.26 (Jang et al., 2022)
Coriolis® μ (with long time monitoring option)	Wetted-wall cyclone (wet)	300	1–10 min (6 h with long-time monitoring option)	50%	Not identified
Isopore™ Membrane Filters 0.4 μm (HTTP03700) with 2-piece conductive filter cassettes (SKC 225–2902)	Filtration (dry)	15	None identified	None identified	0.3
SASS® 3100	Electret filter (dry)	300	Up to days	50%	3.3

Isopore filters were selected as a reference air sampler as they displayed higher physical sampling efficiencies, and better DNA/RNA stability during long-term sampling compared to the more commonly used reference sampler BioSampler, for the test conditions in this study (Supplementary Text 1 in Supplementary File 1). Isopore filters were placed in 2-piece conductive filter cassettes (SKC 225–2902, SKC Inc., PA, USA) with cellulose filter support pads. A rotary vane vacuum pump (SECO SV 1008 C, Busch Vacuum Solutions Norway AS, Drøbak, Norway) was used to achieve a flow rate of 15 L/min, and the flow rate was controlled by a rotameter (Aalborg model P, Aalborg Instruments & Controls, Inc., Orangeburg, NY, USA). SASS used an electret filter (7100–134-232–01, Research International) which consists of a mesh of fibers with electric charge, and had a flow rate of 300 L/min.

VIVAS uses a laminar-flow water condensation particle growth technique to capture aerosol particles at 8 L/min. The temperature settings used for the VIVAS were 5 °C for the conditioner, 45 °C for the initiator, 12 °C moderator, 25 °C for the nozzle, and 15 °C for the sample. Particles were deposited into a 35 mm × 10 mm petri dish with 1.5-ml collection buffer. The liquid cyclone Coriolis had a flow rate of 300 L/min and was tested using the long-time monitoring option with buffer refill during sampling. BioSampler collects particles through liquid impingement and was run continuously with the starting buffer volume of 20 ml for the long-term sampling. The BioSampler was operated by a rotary vane vacuum pump (GAST 1023-V2-G608NGX, Gast Manufacturing Inc., MI, USA) at 12.5 L/min, and the airflow was measured with a mass flow meter (Sierra Top-Trak® model 826, Sierra Instruments, Monterey, CA, USA).

### Aerosol test facility

Air sampler testing was performed in an aerosol test chamber (ATC, Dycor Technologies, Edmonton, AB, Canada) previously described in Dybwad et al. (2014) and Bøifot et al., (2020a, 2020b). Briefly, the ATC was fitted with external heating, ventilation, air conditioning (HVAC), high-efficiency particulate air (HEPA)-filtration systems, two mixing fans, and metrology sensors. An optical particle counter (Grimm 1.108, Grimm Technologies, Douglasville, GA, USA) and an Aerodynamic Particle Sizer (APS 3321, TSI, Shoreview, MN, USA) were used for real-time monitoring of test aerosol concentration and particle size distribution. In addition to the APS, a Fast Mobility Particle Sizer (FMPS 3091, TSI) was used to measure particles < 0.5 μm to control that the total particle concentration in the ATC was below the maximum limit for VIVAS (10^5^ particles/cm^3^). The ATC was kept at 50% relative humidity and a temperature of 23.1 ± 1.5 °C during the trials.

### Test agents and spray solutions

For physical sampling efficiency testing, three different spray solutions were prepared in MQ (MQ water; Purification System, Merck KgaA) water, one for each particle size. The final Uranine concentration (1.08462, Merck KgaA) was 0.5 mg/ml for 0.8 μm particles, 5 mg/ml for 1 μm particles, and 1.5 mg/ml for 3 μm particles.

Two well-characterized model organisms, PA and MS2, were selected as representatives for gram-negative bacteria and viruses, respectively (Bhardwaj et al., 2021; Dybwad & Skogan, 2017). Spray solutions containing PA (ATCC 33243, ATCC, Manassas, VA, USA) were prepared fresh each day. PA was cultured in 30 ml nutrient broth (105,443, Merck KgaA) and incubated overnight (20 h) at 30 °C in an orbital shaking incubator (Corning LSE 71, Corning Inc., Corning, NY, USA) at 200 rpm. The culture was centrifuged at 2500*g* (ThermoFisher Scientific Multifuge X1R, ThermoFisher Scientific, Waltham, MA, USA) for 15 min and the supernatant was removed. For 1 μm particles, the bacterial pellet was resuspended in 30 ml of MQ water, and 2 ml was transferred to 48 ml sterile MQ water with a 0.025 mg/ml final concentration of Uranine. For 3 μm particles, the bacterial pellet was resuspended in 48 ml sterile MQ water together with Uranine at a final concentration of 0.2 mg/ml.

A stock solution of MS2 phage (DSM 13767, DSMZ German Collection of Microorganisms and Cell Cultures GmbH, Braunschweig, Germany) was prepared, and fresh spray solutions were made each day from the stock. In brief, 1.75 ml of an overnight culture of *Escherichia coli* (DSM 5695, DSMZ GmbH) was used to inoculate 50 ml of NZCYM broth (544. NZCYM-medium, DSMZ GmbH) containing 2 mg/l streptomycin (S9137, Merck KgaA) and incubated in an orbital shaking incubator at 37 °C and 200 rpm until the OD_600nm_ was 0.3–0.6. Approximately 1 × 10^10^ PFU MS2 phage was added to the *E. coli* culture and further incubated overnight (20 h) in the orbital shaking incubator at 37 °C and 200 rpm. To the culture, 100 µl lysozyme (25 mg/ml; 1.05281, Merck KgaA) was added and incubated for 30 min (37 °C) before the addition of 100 µl chloroform (1.02444, Merck KgaA) and 100 µl EDTA (0.5 M; 1.08418, Merck KgaA), and allowed to incubate for another 30 min. The culture was centrifuged at 2000*g* to remove cell debris before the supernatant was filtered through a 0.2-μm syringe filter (WHA10462200, Merck KgaA), and the stock was stored at 4 °C. The MS2 stock solution was quantified using a phage plaque assay and the concentration was 4 × 10^10^ PFU/ml. For 1 μm particles, 0.5 ml of MS2 stock solution was diluted in sterile MQ to a final volume of 50 ml together with Uranine at a final concentration of 0.025 mg/ml. For 3 μm particles, 1 ml of the MS2 stock solution was diluted in sterile MQ to a final volume of 40 ml together with Uranine at a final concentration of 0.5 mg/ml.

### Aerosol generation

For physical sampling efficiency testing with Uranine, the mass median aerodynamic diameter (MMAD) was 0.8, 1.3, and 3.4 μm, and the MMAD for aerosols containing MS2 or PA was 1.5 and 3.4 μm. Hereafter, referred to as 1 and 3 μm particles. The particle size distributions were calculated based on APS 3321 measurements from at least five separate experiments as mean (± standard deviation) of numerical median aerosol diameter (NMAD; μm), MMAD (μm), and geometric standard deviation (GSD; unitless), and can be found in Table S1 in Supplementary File 2 with the particle concentration (particles/ml). The particle background in the empty ATC had an NMAD of 0.7–0.8 μm and a concentration of 0.1–0.2 particles/ml, and during long-term sampling, the NMAD was 0.8 μm with a concentration of 0.5–0.9 particles/ml. When instruments were running inside the ATC, small particles were generated, and this is reflected in the slightly higher particle background during long-term sampling compared to the empty ATC.

**Fig. 1:**
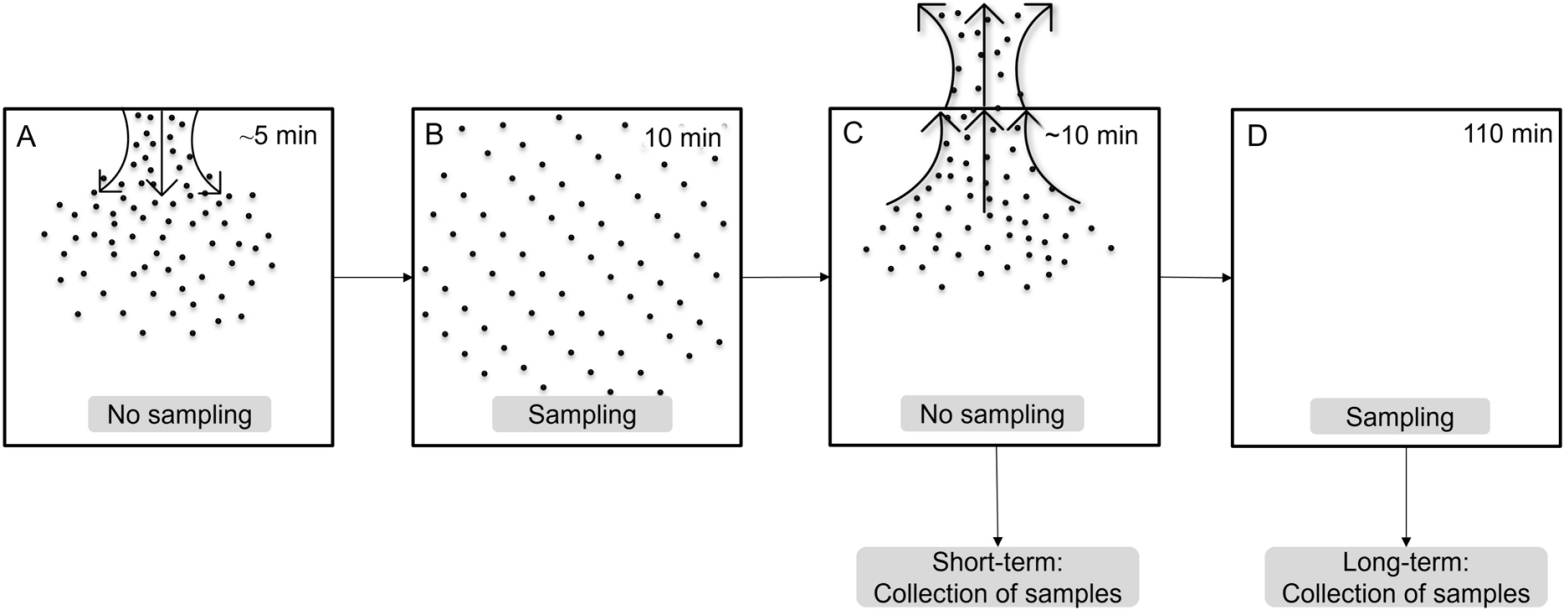
The different processes in the ATC. **A** Aerosolization and homogenization (~ 5 min) before sampling, **B** sampling initiated (10 min), **C** sampling stopped and the ATC purged for particles, for short-term sampling the samples were collected, **D** for long-term sampling the sampling continued in an empty chamber for 110 min before samples were collected

Aerosol particles of Uranine with an MMAD of 0.8 μm and 1 μm were generated using a Hudson RCI 1710 Up-Draft nebulizer (Medline International B.V., Arnhem, Netherlands) propelled with N_2_ gas. Aerosol particles with an MMAD of 1 μm (MS2 and PA) and 3 μm (Uranine, MS2, and PA) were produced with a 120-kHz and 48-kHz ultrasonic atomizer nozzle (Sono-Tek, Milton, NY, USA) respectively, and powered with 2 W by an ECHO multiband ultrasonic generator (Model 06–05–00330, Sono-Tek). The spray solution was loaded into 50-ml Luer lock syringes placed in a syringe infusion pump (Model 997E, Sono-Tek), and the ultrasonic atomizer was fed with 1–1.5 ml/min for 3–4 min. After dissemination, the ATC was homogenized with the internal mixing fans for 1 min before initiating sampling (Fig. 1). The mixing fans continued to operate throughout the experiments to create stirred settling sampling conditions. Appropriate instrument settings for the ATC and its subsystems were determined during pre-study experiments and kept static throughout the study. The total amount disseminated was adjusted for each test setting such that the total aerosol biomass collected in 10 min was within the quantitation limits of the quantitative PCR (qPCR) assays for all air samplers. The airflow inside the ATC has previously been measured and shown to be < 0.7 ms^−1^ in all sampling locations (Bøifot et al., 2020a, 2020b).

### Aerosol collection

All air samplers were positioned inside the ATC, except the VIVAS which was placed underneath with a conductive tube extending in a straight vertical line into the ATC. All air sampler inlets were located 20 cm above the ATC floor.

For sampling with Uranine, five trials for each particle size (0.8, 1, 3 μm) were performed with simultaneous collection with all air samplers for 10 min. Between each trial, the ATC was purged (~ 10 min) before air samples were recovered. Isopore and SASS filters were placed in 10 ml PBS (P4417, Merck KgaA) with 0.05% Trition-X-100 (Merck 11,869, Merck KgaA) and 0.005% Antifoam-A (A5633, Merck KgaA; PBSTA) and vortexed (20 s) for extraction of particles. Coriolis, VIVAS, and BioSampler used MQ water as a collection buffer for the physical sampling efficiency tests with Uranine. Autoclaved MQ water was used as a refill buffer in VIVAS and injected into the growth tube wicks at 20 μl/min. MQ water was also used as a refill buffer in Coriolis. After sampling, the end volumes for the liquid samples were recorded, and samples were kept in the dark at 4 °C before fluorometric analysis.

For bioaerosols, simultaneous sampling with the reference sampler was conducted at least 5 times for each particle size (1 and 3 μm) and test agent (MS2 and PA). Two different sampling times were used, 10 min (short-term sampling) and 2 h (long-term sampling). The short-term sampling acted as a reference to compare the effect of sampling stress during long-term sampling. For long-term sampling, there was 10 min of active sampling before the ATC was purged and the air samplers could continue sampling clean air for 110 min, in total 2 h (Fig. 1). Filter extraction was performed as described for Uranine collection. For bioaerosol sampling, PBS was used as a collection buffer for Coriolis, VIVAS, and BioSampler, while MQ water was used as a refill buffer in Coriolis and VIVAS as described for Uranine collection. Similar to Uranine collection, end volumes for the liquid samples were recorded. Since VIVAS had a lower end volume than the other air samplers, the entire end volume was transferred to 7.5 ml PBS before aliquots were taken for nucleic acid extraction. All samples were vortexed for 20 s before an aliquot was transferred to 10 ml NucliSENS lysis buffer (BioMérieux, Marcy-l’Étoile, France). For samples containing MS2, a 4 ml aliquot was used for all samplers. For PA, a 4 ml aliquot was used for VIVAS, BioSampler, and isopore filters. For the high-volume samplers (SASS and Coriolis), 0.4 ml was used, as 4 ml resulted in concentration above the limit of quantification for the qPCR assay. Lysis buffer samples for nucleic acid extraction were stored at 4 °C, or at − 80 °C if samples were not processed within 3 days.

To investigate the potential for sample-to-sample cross-contamination, samples were collected in an empty ATC following the same conditions as described above, and the results showed negligible traces of contamination (> 100-fold less than during aerosol experiments) with test agent-specific qPCR assays.

### Nucleic acid extraction and qPCR

Nucleic acid extraction was performed with the NucliSENS Magnetic Extraction Reagent Kit (BioMérieux, Marcy-l’Étoile, France). The manufacturer’s protocol was followed but with 90 μl magnetic beads instead of 50 μl. Nucleic acids were quantified with qPCR using test agent-specific primers and probes (Table S2 in Supplementary File 2; Invitrogen, Waltham, MA, USA) for MS2 (O’Connell et al., 2006) and PA (Braun-Kiewnick et al., 2012). The MS2 assay used the RNA Virus Master (Cat. No. 06754155001, Roche Diagnostics, Oslo, Norway) with a total reaction volume of 20 μl, with 5 μl sample and a final concentration of 0.5 μM forward and reverse primer and 0.25 μM probe. The amplification was performed on a LightCycler 96 (Roche) starting with reverse transcription at 50 °C for 10 min, and followed by 45 cycles at 95 °C for 5 s and 60 °C for 30 s. The PA assay was performed in a 20 μl volume using SYBR Green Master (Cat. No. 04707516001, Roche) with 2-μl sample and a final concentration of 0.5 μM of each primer. Amplification was performed on a LightCycler 96 with 10 min preincubation at 95 °C, followed by 40 cycles of 95 °C for 10 s, 60 °C for 20 s, and 72 °C for 30 s. Standard curves were created by serial dilution of MS2 RNA and PA DNA.

### Fluorimeter analysis

Uranine concentrations were measured using a FLUOStar Optima microplate fluorimeter (BMG Labtech, Offenberg, Germany). All samples were vortexed for 20 s before aliquots were taken for analysis. Due to the high flow rate of SASS and Coriolis, these samples were diluted tenfold. Samples from filter and liquid samplers were obtained in different buffers, and to gain an equal concentration of Triton X-100 before fluorescence measurement, 100 μl of sample was mixed with 100 μl of either PBS or PBSTA. Thereafter, 200 μl 0.1 M Tris-base buffer pH 9.5 (Sigma-Aldrich, St. Louis, MO, USA) was added to each sample and mixed well before 100 μl triplicates were measured using Corning 3915 black 96-well microplates (Sigma-Aldrich). To generate a standard curve, Uranine was serially diluted in the same buffer as the samples.

### Calculation and statistical analysis

**Fig. 2:**
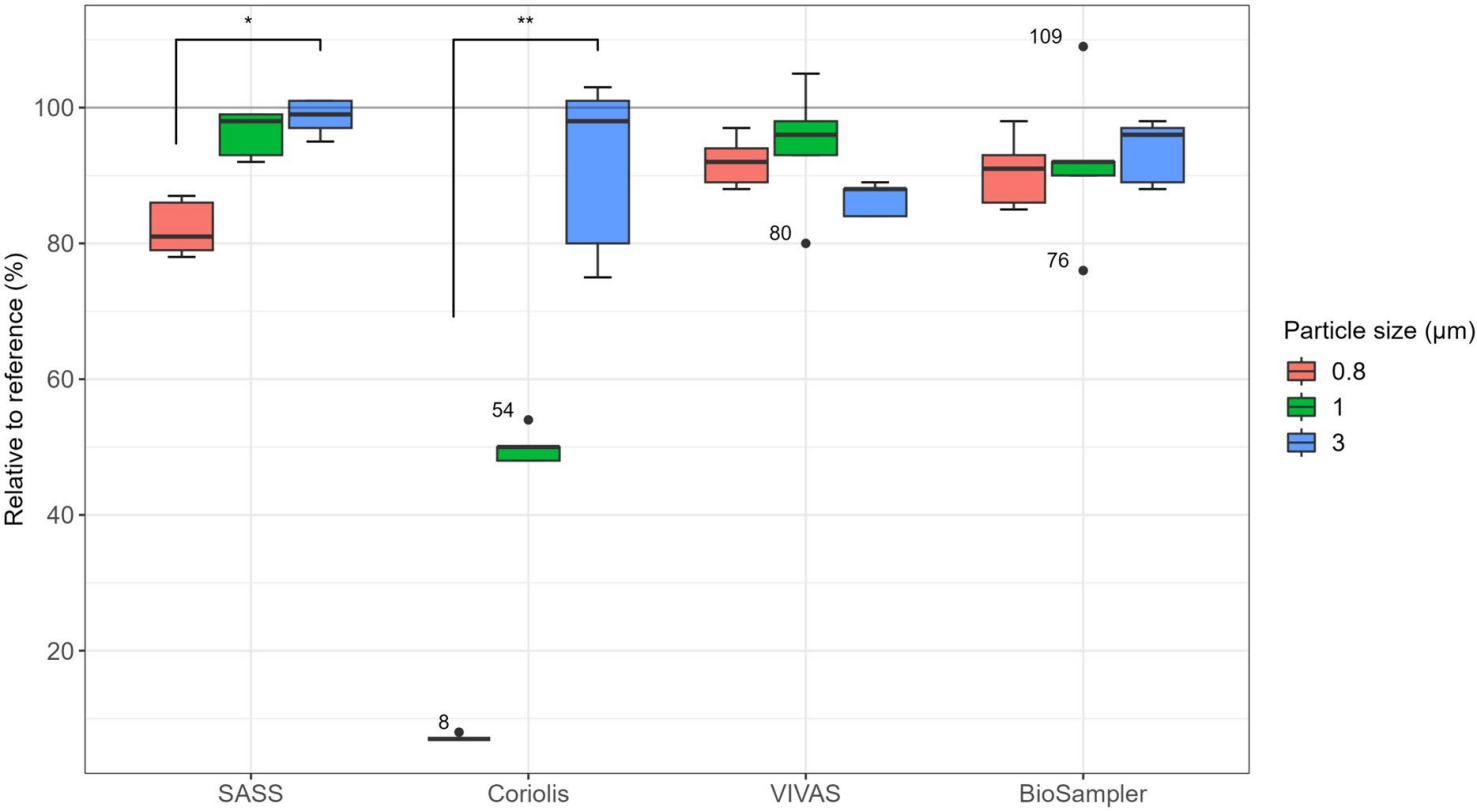
Physical sampling efficiencies relative to the reference sampler (%) were determined with Uranine at 0.8, 1, and 3 μm (MMAD), for SASS, Coriolis, VIVAS, and BioSampler. Percentages above or below 100% indicate that the performance was better or worse than for reference, respectively. Experiments were repeated five times for each particle size. The Kruskal–Wallis test with post hoc Dunn’s test was used to identify significance between particle sizes which is indicated with * (*P* < 0.05) or ** (*P* < 0.005)

**Fig. 3:**
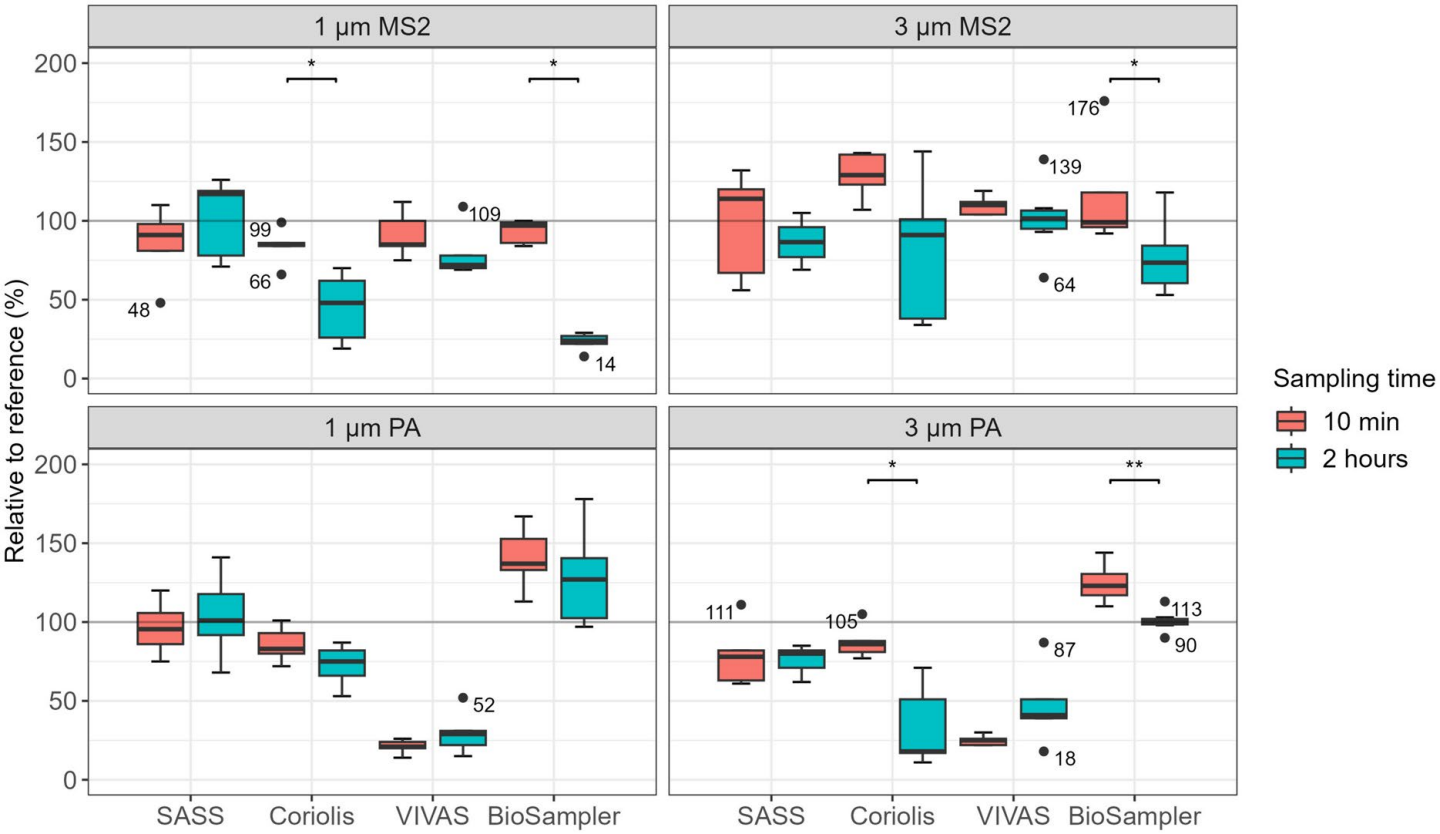
Sampling efficiencies for MS2 and PA for SASS, Coriolis, VIVAS, and BioSampler for 1 and 3 μm particles relative to the reference (%). Percentages above or below 100% indicate that the performance was better or worse than for reference, respectively. Two different sampling times (10 min and 2 h) were used to investigate nucleic acid stability during long-term sampling. The active sampling time was 10 min, and for long-term sampling, it was followed by 110-min sampling in clean air. Experiments were repeated at least 5 times for each condition and sampler. A Mann–Whitney test was used to identify significant changes in relative concentration between 10 min and 2 h, and significance levels are indicated with * (*P* < 0.05) or ** (*P* < 0.005)

Results were expressed as μg Uranine/m^3^ of air (physical sampling efficiency) or genome copies/m^3^ of air (nucleic acid stability) to compensate for the different flow rates and made relative to isopore filters (reference). SPSS 29.0 (IBM SPSS Statistics) was used to analyze the results. An independent samples Kruskal–Wallis test was performed for pairwise comparison of air samplers and particle sizes. Post hoc Dunn’s test was performed in cases where the Kruskal–Wallis test was significant. An independent samples Mann–Whitney *U* test was performed to investigate the significance level between 10 min and 2 h. Bonferroni correction was used to correct *P*-values for multiple comparisons. The significance level was set to < 0.05. Boxplots (Figs. 2 and 3) were created in R using Tidyverse and ggsignif, while boxplots in supplementary were created in SPSS 29.0.

## Results

### Physical sampling efficiency

Physical sampling efficiencies relative to the reference sampler for 0.8, 1, and 3 μm particles were determined for the evaluated air samplers using Uranine (Fig. 2 and Table S3 in Supplementary File 2).

SASS had a significantly higher sampling efficiency for 3 μm particles compared to 0.8 μm (99 ± 2% vs 82 ± 4%, *P* = 0.007), while no significant difference was found between 1 μm (96 ± 3%) and the two other particle sizes. Coriolis showed a significantly lower sampling efficiency for 0.8 μm particles compared to 3 μm particles (7 ± 0.3% vs 91 ± 11%, *P* = 0.001), while no significant difference was found between 1 μm (50 ± 2%) and the two other particle sizes. VIVAS showed no significant difference in sampling efficiency for the three particle sizes (0.8 μm: 92 ± 3%, 1 μm: 94 ± 8% and 3 μm: 86 ± 2%), nor did BioSampler (0.8 μm: 91 ± 5%, 1 μm: 92 ± 11% and 3 μm: 94 ± 4%).

A nonparametric Kruskal–Wallis test showed that there were no significant differences between the air samplers for 3 μm particles (*P* = 0.092), while significant differences were found for 0.8 (*P* = 0.002) and 1 μm (*P* = 0.008) particles. A post hoc pairwise comparison of the different air samplers showed that for 0.8 μm particles there was a significant difference between Coriolis and VIVAS (7 ± 0.3% vs 92 ± 3%, *P* = 0.003), and Coriolis and BioSampler (7 ± 0.3% vs 91 ± 5%, *P* = 0.015). For 1 μm particles, there was a significant difference between Coriolis and SASS (50 ± 2% vs 96 ± 3%, *P* = 0.011), and Coriolis and VIVAS (50 ± 2% vs 94 ± 8%, *P* = 0.027).

In summary, there were no significant differences (*P* ≥ 0.301) between BioSampler, SASS, and VIVAS for 0.8 and 1 μm particles, and for 3 μm particles, there was no significant difference (*P* = 0.092) between any of the air samplers. However, Coriolis showed significantly lower sampling efficiencies (for 0.8 and 1 μm particles) compared to the other air samplers.

### Nucleic acid stability

Aerosols containing test agents (MS2 and PA) were generated at two different particle sizes (1 and 3 μm), totaling four test conditions, to investigate the stability of nucleic acids during long-term sampling (Fig. 3 and Table S3 in Supplementary File 2).

For 1 μm MS2 particles, there was a significant decrease from 10 min to 2 h for Coriolis (84 ± 11% vs 45 ± 20%, *P* = 0.016) and similarly for BioSampler (93 ± 6% vs 23 ± 5%, *P* = 0.008), while no significant difference was observed for SASS (86 ± 21% vs 102 ± 23%, *P* = 0.421) and VIVAS (91 ± 13% vs 80 ± 15%, *P* = 0.151). For 3 μm MS2 particles, there was a significant decrease after 2 h for BioSampler (116 ± 31% vs 77 ± 21%, *P* = 0.030), while no significant difference was observed for SASS (98 ± 31% vs 87 ± 13%, *P* = 0.662), Coriolis (129 ± 13% vs 82 ± 41%, *P* = 0.151) and VIVAS (110 ± 6% vs 101 ± 22%, *P* = 0.177). For 1 μm PA particles, there was no significant difference between 10 min and 2 h for any of the air samplers, SASS (97 ± 14% vs 103 ± 21%, *P* = 0.481), Coriolis (86 ± 10% vs 72 ± 12%, *P* = 0.222), VIVAS (21 ± 4% vs 30 ± 12%, *P* = 0.222), and BioSampler (140 ± 17% vs 129 ± 28%, *P* = 0.247). For 3 μm PA particles, there was a significant decrease for Coriolis (87 ± 9% vs 34 ± 24%, *P* = 0.008) and BioSampler (124 ± 11% vs 101 ± 6%, *P* = 0.002), while no significant difference was observed for SASS (79 ± 18% vs 76 ± 8%, *P* = 0.841) and VIVAS (25 ± 3% vs 47 ± 23%, *P* = 0.151).

Only Coriolis and BioSampler, both using a collection principle that leads to loss of collection buffer, showed a significant decrease in genome copies/m^3^ relative to the reference after 2-h sampling. Coriolis also had a significant decrease for all test conditions in Uranine concentration (Fig. S1 in Supplementary File 2) after 2 h for 1 μm MS2 (94 ± 13% vs 36 ± 22%, *P* = 0.008), 3 μm MS2 (108 ± 14% vs 64 ± 33%, *P* = 0.032), 1 μm PA (102 ± 8% vs 66 ± 6%, *P* = 0.008), and 3 μm PA (97 ± 14% vs 28 ± 14%, *P* = 0.008). BioSampler, which also showed a decrease in genome copies/m^3^, did not display a similar decrease in Uranine concentrations. A new set of experiments was therefore conducted with Coriolis, using PBS spiked with Uranine as a collection buffer, and running the instrument for up to 2 h (Supplementary Text 2 in Supplementary File 1). Coriolis showed a significant decrease in Uranine concentration after 10 min, 1 h, and 2 h, suggesting that Uranine was lost during operation of the instrument. Rinsing the air inlet and metal flow cane of the instrument with water reduced variability in the spike results. This suggests that rinsing removed contamination in the air inlet and metal flow cane that could otherwise contaminate the sample through backflow.

It was observed that 1 μm PA showed stable genome copy yields relative to the reference for all air samplers, also for Coriolis, which had shown a significant decrease in Uranine concentration. The characterization of the reference sampler (Supplementary Text 1 Supplementary File 1) showed that there was a decrease in DNA for 1 μm PA from 10 min to 2 h. Therefore, raw values (genome copies/m^3^) were used to identify if there was a decrease in genome copies after 2 h (Table S4 and S5 in Supplementary File 2) for all air samplers. There was a significant decrease in genome copies from 10 min to 2 h for 1 μm PA for the reference (4.21 × 10^7^ vs 2.72 × 10^7^, *P* = 0.013) and a non-significant decrease in genome copies for SASS. Coriolis and BioSampler also had a significant decrease in genome copies from 10 min to 2 h for 1 μm PA based on raw values, while VIVAS showed a stable concentration.

VIVAS showed a notable difference in sampling efficiency between MS2 and PA. However, the Uranine concentration (tracer) did not differ significantly (*P* ≥ 0.329) between MS2 and PA, suggesting that the experiments and the instrument had worked successfully. Theoretical calculations were performed to investigate if there was an uneven distribution of PA and Uranine particles which could give rise to differential sampling (Supplementary Text 3 in Supplementary File 1). Theoretical calculations showed that every 3 μm PA particle would contain Uranine and several viable PA cells. For 1 μm PA, all particles would contain Uranine, while only 10% of the particles would contain both viable PA and Uranine. The low fraction of PA particles could potentially lead to differential sampling, but as this was only observed for 1 μm PA it was unlikely that differential sampling was an issue. This led to additional experiments examining the potential adhesion of PA cells or cell fragments to the petri dish in which VIVAS samples were deposited (Supplementary Text 4 in Supplementary File 1), but no signs of adhesion were observed for any of the plasticware tested.

## Discussion

In this study, we evaluated four air samplers (SASS 3100, Coriolis μ with long-time monitoring option, BioSpot-VIVAS 300P, and SKC BioSampler) for physical sampling efficiency and nucleic acid stability during long-term sampling. All air samplers, except Coriolis, achieved high physical sampling efficiencies (> 80%) for all evaluated particle sizes (0.8, 1, and 3 μm). Our results showed that BioSampler (impingement) and Coriolis (wetted-wall cyclone) had a reduction of DNA (PA) and RNA (MS2) after 2-h sampling, while SASS (electret filter) only experienced a reduction of DNA for 1 μm PA particles. VIVAS showed stable RNA and DNA quantities after 2-h sampling but had a relatively poor sampling efficiency for PA compared to the reference sampler.

Physical sampling efficiency is a measure of how efficiently an air sampler collects particles and how efficiently collected material can be recovered from a sample. As mentioned, the physical sampling efficiencies were high for all test conditions and samplers (> 80%) except Coriolis, and the efficiencies were as expected based on previous reports and manufacturer-supplied specifications and test data (Aerosol Devices Inc., n.d.; Bøifot et al., 2020a, 2020b; Dybwad et al., 2014; Kesavan et al., 2010; SKC Inc., n.d.-a). Coriolis had a lower physical sampling efficiency (7%) than expected for 0.8 μm particles based on the specified D50 for < 0.5 μm (50%) (Bertin Technologies, 2022). However, Coriolis had a high physical sampling (> 80%) for 3 μm particles and as expected for 1 μm particles (50%) based on previous reports (Dybwad et al., 2014). While the physical sampling efficiency for Coriolis based on Uranine was 50 ± 2% for 1 μm particles, the tracer (Uranine) used in experiments with 1 μm MS2 and PA showed higher sampling efficiencies with 94 ± 13% and 102 ± 8%, respectively. This was likely caused by a difference in the aerosol generation method which resulted in a lower NMAD for physical sampling efficiency experiments (Hudson nebulizer) compared to that of MS2 and PA experiments (120 kHz Sono-Tek), though the MMAD was similar. Differences in aerosol generation methods could also explain the unexpectedly low sampling efficiency for 0.8 μm particles compared to the D50 stated by the manufacturer.

When investigating the nucleic acid stability during long-term sampling, only VIVAS showed stable DNA and RNA quantities after 2 h compared to 10 min for all test conditions. On the contrary, the liquid samplers Coriolis and BioSampler displayed reduced DNA and RNA stability relative to the reference for all conditions. However, for 1 μm PA, both Coriolis and BioSampler displayed stable concentrations relative to the reference after a 2-h sampling, but the apparent stability was caused by a significant reduction (genome copies/m^3^) in the reference sampler, which was not observed for the other test conditions. Based on raw values (genome copies/m^3^), Coriolis and BioSampler had a significant reduction after 2 h for 1 μm PA, and a reduction was also observed for SASS, though not significant. The observed reduction of DNA in 1-μm PA experiments for filter samplers is likely a result of desiccation and degradation of DNA during long-term sampling. This was not an issue for 3 μm PA but microorganisms in smaller particles can be more exposed to desiccation than larger particles (Lighthart & Shaffer, 1997). No reduction in RNA for filter samplers was observed for either 1 or 3 μm MS2, but it has previously been suggested that due to the small size of MS2 (27 nm) even particles from 100 to 450 nm provide a shielding effect for survival of MS2 (Zuo et al., 2014). The results show that reduction of DNA is an issue with filter sampling for certain conditions during long-term sampling. In real-world sampling, this would lead to a non-representative sample by underestimating PA compared to MS2. However, this study only included two test agents and did not include gram positives or spores which are considered to be more resistant to sampling stress. Further studies are needed to understand if this is a widespread issue for smaller particles containing microorganisms and, by that, the impact it may have on microbiome studies.

Coriolis did not only show a reduction in genome copies, a significant decrease in Uranine concentration was observed for all test conditions. Additional spike experiments with Uranine showed a significant reduction in Uranine concentrations even after 10 min of running the Coriolis. The loss of Uranine can be attributed to evaporation/reaerosolization as no evidence of photobleaching was found for the duration and environmental conditions used in the spike experiments. Rufino de Sousa et al. (2020) have previously shown that reaerosolized material can be deposited internally in Coriolis. The Uranine concentration in spike samples was highly variable after 2 h, but the variability was reduced when the air inlet and metal flow cane was rinsed with water in between every run. As large volumes of collection buffer evaporate during long-term sampling with Coriolis (Tseng et al., 2020), there are concerns that this liquid may condense in the interior walls of the air inlet and flow cane. This can cause a random backflush into the sampling cone, which can contribute to cross-contamination between samples and could explain the large variations observed for Coriolis. Based on our findings, we would recommend cleaning or rinsing between each run, especially for long-term sampling, and not each day or between each controlled room as stated in the manufacturer’s manual. While Coriolis displayed a decrease in both Uranine and genome copies, BioSampler only showed a decrease in genome copies and had relatively stable Uranine concentrations for all test conditions. Reaerosolization from BioSampler has been reported several times (Lin et al., 2000; Riemenschneider et al., 2010), but Han and Mainelis (2012) found that the largest loss of material was internally in the BioSampler. Lemieux et al. (2019) have shown that different bacteria can reaerosolize at different rates in the BioSampler. Differential reaerosolization could explain the difference in stability between Uranine and MS2/PA in BioSampler, but based on our results we cannot conclude what mechanisms (reaerosolization, internal loss, or degradation) contributed to the loss of MS2 and PA. It is also important to note that sampling buffers can impact on evaporation rates and by that reaerosolization. For BioSampler it is recommended to use mineral oil or glycerol to avoid evaporation during long-term sampling but can affect downstream molecular processes (Pan et al., 2018; SKC Inc., n.d.-b). Loss of collected material from liquid air samplers is a recognized problem and should be taken into account if used in microbiome studies where representative samples are essential.

An interesting finding in this study was the difference in sampling efficiency (relative to reference) observed between PA and MS2 with VIVAS, where MS2 had sampling efficiencies around 100% and PA around 20% for short-term sampling. Translated into real-world sampling, this would result in a non-representative sample, where PA was underestimated. The MS2 results were as expected based on other studies, which have shown that the condensation growth principle employed by VIVAS has high sampling efficiencies for MS2 and viruses when compared to other air samplers (Degois et al., 2021; Jiang et al., 2016; Raynor et al., 2021). Despite the low sampling efficiency for PA, Uranine was stable between all experiments, suggesting that the sampler had operated correctly. Other factors to explain the difference in sampling efficiency were investigated, such as VIVAS’ upper particle concentration limit, differential sampling between PA and Uranine, and adhesion of PA to the collection petri dish. However, none of the investigated factors could explain the observed result. A limitation of the spike experiments investigating adhesion is that aerosolization may impact the surface properties of the particles/microorganisms which is not easily reproduced. Few published studies have compared condensation growth to other sampling principles using bacteria. Nieto-Caballero et al. (2019) looked at the stability of *Bacillus subtilis* using the SpotSampler (based on the same condensation growth technique as VIVAS) and found a small decrease in 16S rRNA gene copy numbers after a 50-min sampling, which corresponds well with this study. Nieto-Caballero (2021) compared SpotSampler, BioSampler, and polycarbonate filters (isopore filters), and showed that the SpotSampler performed better (judged by 16S rRNA gene copy number/m^3^) than the two other samplers for *B. subtilis*. Differences in experimental factors (e.g., collection buffer, filter extraction, test agents, instruments, and DNA isolation) may have contributed to the opposite conclusions, but there are not enough experimental details available for a thorough comparison. Therefore, further studies are warranted to identify the cause of the discrepancy. It would be interesting to compare VIVAS using other bacterial and fungal species, and collection directly into a nucleic acid preservative as this would enhance the preservation of collected samples.

We did not explore the effect of different collection principles on real-world samples in this study, but previous studies have compared microbial diversity for some of the air samplers evaluated in this study (SASS, Coriolis, BioSampler, and isopore filters). Real-world data shows conflicting results regarding the comparability of the air samplers. Mbareche et al. (2018) concluded that Coriolis did not cover most of the bacterial and fungal diversity found with SASS. Lemieux et al. (2019) found that SASS and isopore filters had statistically higher species richness than Coriolis and BioSampler and that the two filter samplers had a comparable bacterial diversity (top 20) that was different from that found by Coriolis and BioSampler. On the other hand, Luhung et al. (2021) concluded that SASS and Coriolis displayed comparable microbial diversity based on the top 40 most abundant organisms. While Mbareche et al. (2018) and Lemieux et al. (2019) have used an almost identical methodology and sampled in a similar environment, Luhung et al. (2021) have used a different sample processing, sequencing, and bioinformatics analysis scheme, which may have contributed to the conflicting results. The effect different protocols have on the microbiome should not be underestimated. There is a need to further characterize and harmonize air sampler selection and experimental protocols for microbiome studies so results can be leveraged across studies, to advance our understanding of the air microbiome.

## Conclusion

Increasing air sampling time to collect enough biomass for sequencing studies can come at an expense. The stability of microorganisms and their nucleic acids during long-term sampling is a concern as representative sample collection is crucial for the validity of microbiome studies. By challenging different air samplers with viruses and bacteria, we studied the stability of nucleic acids during long-term sampling to improve our understanding of how this strategy can affect real-world microbiome studies. We hypothesized that nucleic acid yields would decrease after a 2-h sampling, and this was the case for all test conditions (1 and 3 μm PA, and 1 and 3 μm MS2) for liquid-based collection with BioSampler and Coriolis, and for 1 μm PA for filter-based collection with SASS and isopore filters. VIVAS displayed stable yields for long-term sampling, but with lower sampling efficiency for PA compared to MS2. All air samplers included in this study were associated with some limitations that would affect aerosol microbiome studies. Long-term sampling with filters and sampling with condensation growth would, based on our result, collect a non-representative sample, while valuable biomass would be lost from liquid-based air samplers (e.g., through reaerosolization). Our results support that there are fundamental differences between the collection principles which can manifest as differences in microbial diversity. This shows the importance of considering the bias introduced by air sampling when selecting air samplers for microbiome studies, and when interpreting microbiome data. As it stands, no air sampler is perfect, and new investigations are needed to understand the mechanisms behind the bias and how they can be overcome to unlock the true microbial diversity.

### Supplementary Information

Below is the link to the electronic supplementary material.

Supplementary file1 (DOCX 48.4 KB)

Supplementary file2 (XLSX 395 KB)

## Data Availability

All data generated and analyzed during this study are included in this published article and its supplementary information files.
