# Supplementary material for: Sampling efficiency and nucleic acid stability during long-term sampling with different bioaerosol samplers
Source: Environ Monit Assess. 2024 May 25;196(6):577. doi: 10.1007/s10661-024-12735-7 (PMC11127824; doi:10.1007/s10661-024-12735-7)
Supplement: Supplementary file 1 — Supplementary file1 (DOCX 48.4 KB) [file 10661_2024_12735_MOESM1_ESM.docx]

**Supplementary file 1 - Sampling efficiency and nucleic acid stability during long-term sampling with different bioaerosol samplers**

Kari Oline Bøifot^1,2^, Gunnar Skogan^1^ and Marius Dybwad^1,2^

1) Norwegian Defence Research Establishment, P O Box 25, NO-2027 Kjeller, Norway

2) Department of Analytical, Environmental and Forensic Sciences, King’s College London, 150 Stamford Street, London SE1 9NH, UK

**Supplementary Text 1. Selection of reference sampler.**

**Aim**

Characterization and selection of reference sampler.

**Background**

A reference sampler is often used in aerosol chamber experiments to compare the performance of different air samplers. Air samplers such as AGI-30, BioSampler, gelatin filters, and polycarbonate (isopore) membrane filters have been used in the past. During long-term sampling, the BioSampler’s collection buffer will evaporate which could affect its performance as a reference sampler, while isopore filters have previously shown high and stable physical sampling efficiencies. Therefore, the performance of BioSampler and isopore filters were compared for the test conditions used in this study.

**Method**

Physical sampling efficiency for isopore filters relative to BioSampler was investigated using Uranine at three different particle sizes (0.8, 1, and 3 µm) as described in the “Materials and methods” section.

The stability during long-term biological sampling was investigated for isopore filters and BioSampler by comparing the ratio between 2 hours and 10 minutes for Uranine (µg/m^3^), MS2 and PA (genome copies/m^3^).

**Results**

The physical sampling efficiency of isopore filters was around 10% higher than for BioSampler for 0.8, 1, and 3 µm Uranine particles (Supplementary Text 1 Table 1) which is comparable to previous results (Bøifot et al., 2020).

**Supplementary Text 1 Table 1.** Physical sampling efficiency for isopore filters relative (%) to BioSampler for three particle sizes using Uranine. Experiments were repeated five times.

|  | 0.8 µm | 1 µm | 3 µm |
| --- | --- | --- | --- |
| Isopore | 110±6 | 110±13 | 107±5 |

BioSampler showed a significant reduction in DNA/RNA concentration from 10 minutes to 2 hours for three out of four test conditions, while isopore filters only had a significant reduction in one test condition (Supplementary Text 1 Table 2). The ratio of Uranine was overall more stable for the different test conditions for isopore filters compared to BioSampler.

**Supplementary Text 1 Table 2.** The ratio between 2 h and 10 min of MS2, PA, and Uranine for isopore filters and BioSampler. Experiments were conducted at least five times. Significant results with Mann-Whitney *U* test are indicated with * (*P* ≤ 0.05).

| Ratio: 2 h/10 min | | MS2 | | PA | |
| --- | --- | --- | --- | --- | --- |
| Sampler | Particle size | 1 µm | 3 µm | 1 µm | 3 µm |
| **Isopore filters** | Uranine | 0.97 | 1.15 | 0.96 | 1.06 |
|  | DNA/RNA | 1.04 | 1.10 | 0.65* | 0.96 |
| **BioSampler** | Uranine | 0.96 | 1.44* | 0.83 | 1.19 |
|  | DNA/RNA | 0.26* | 0.73* | 0.56* | 0.80 |

**Conclusion**

Isopore filters showed higher physical sampling efficiency and higher stability between 10 min and 2 h for Uranine and DNA/RNA concentrations than BioSampler. Isopore filters were therefore considered to be the most suitable reference sampler for long-term sampling for the test conditions in this study. However, for one test condition, the isopore filter experienced significant reduction which should be taken into account when interpreting the results.

**Supplementary Text 2.** **Uranine stability in Coriolis µ.**

**Aim**

The aim was to calculate the loss of Uranine in the Coriolis collection buffer after running the instrument for up to 2 h.

**Methods**

Uranine was added to PBS with a final concentration of 0.1 µg/ml. The Coriolis sampling cone was filled with 15 ml PBS w/Uranine and the instrument was allowed to run for 10 min, 1 h, and 2 h with refill (MQ) to maintain the buffer level. To verify that the fluorescence of Uranine was not photobleached, a closed sampling cone with PBS w/Uranine was placed next to the Coriolis for the entire run. A control sample was collected at the start of each run. After completing a run, the buffer level was measured, and an aliquot was collected for analysis. This was repeated at least 5 times for each time setting. The PBS and samples were stored dark at 4°C. Samples were treated and measured as described in “Fluorimeter analysis” under the materials and methods section of the main paper. The loss was calculated based on measured concentrations in the control samples. A Mann-Whitney *U* test was performed for each time setting to identify if there was a significant decrease.

**Results**

Coriolis showed an increasing loss of Uranine relative to the control samples with increased sampling time, Supplementary Text 2 Figure 1. It was observed that the results were highly variable after 2 h. Additional experiments were performed where the sampling inlet and metal flow cane were rinsed with water between every run. This appeared to reduce some of the variability after 2-h sampling. There was a significant decrease for all time settings compared to the control, and the average concentration left in the sampling cone relative to the control sample was 93±3% (*P* = 0.008) after 10 min without rinse, 77±2% (*P* = 0.008) after 1 h with rinse, 52±18% (*P* = 0.009) after 2 h with rinse and 46±33% (*P* < 0.001) after 2 h without rinse.


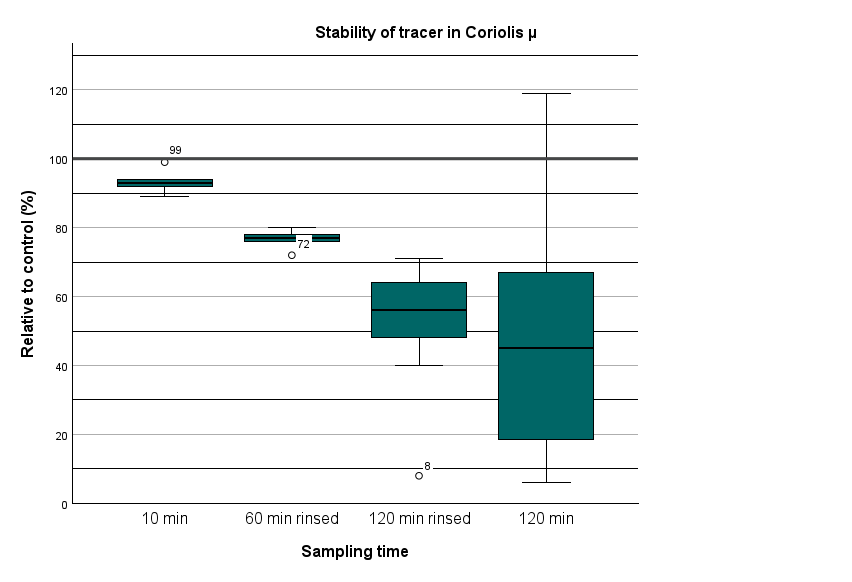


**Supplementary Text 2 Figure 1.** Uranine concentrations relative to control sample (%) for Coriolis. Collection buffer (PBS) was spiked with Uranine to estimate the loss of Uranine during the operation of the instrument at different time settings. Experiments where the sampling inlet and metal flow cane were rinsed with water in-between runs are indicated as “rinsed”.

**Supplementary Text 3. Particle content.**

**Aim**

Calculate the theoretical particle content based on PA and Uranine concentrations in the spray solutions.

**Methods**

To calculate the theoretical particle content in particles generated with 48 and 120 kHz Sono-Tek nozzles, the theoretical median droplet diameter was used for each nozzle (38 and 18 µm, respectively). The volume of each droplet was calculated and the number of droplets in 1 ml of water-based spray solution was estimated. The concentration of each component (PA, MS2, and Uranine) in the spray solution was divided by the number of particles to decide the number of cells or µg Uranine in each droplet.

**Results**

The number of droplets per ml of spray solution for 48 and 120 kHz was 3.48×10^7^ and 3.27×10^8^, respectively. The theoretical results (Supplementary Text 3 Table 1) show that only 10% of 1 µm PA particles would contain PA, while each 3 µm PA particle would contain 17 PA cells per particle. For MS2 particles, there was 1 MS2 phage per 1 µm particle and 29 MS2 per 3 µm particle.

**Supplementary Text 3 Table 1.** Theoretical droplet content for each test condition.

| Particle type | Particle size (µm) | Concentration/ml in spray solution | | Droplets/ml of spray solution | Theoretical content per particle | |
| --- | --- | --- | --- | --- | --- | --- |
|  |  | Cells/phages | Uranine (µg) |  | Cells/phages | Uranine (µg) |
| PA | 1 | 4×10^7^ | 25 µg | 3.27×10^8^ | 0.12 | 7.7×10^-8^ |
| PA | 3 | 6×10^8^ | 200 µg | 3.48×10^7^ | 17.2 | 5.7×10^-6^ |
| MS2 | 1 | 4×10^8^ | 25 µg | 3.27×10^8^ | 1.22 | 7.7×10^-8^ |
| MS2 | 3 | 1×10^9^ | 500 µg | 3.48×10^7^ | 28.7 | 1.4×10^-5^ |

**Supplementary Text 4.** **Adhesion of PA to plasticware.**

**Aim**

Identify if PA adheres to plastic surfaces.

**Methods**

A solution of PA and Uranine was created as described in “Test agents and spray solutions” for 3 µm PA spray solutions in the materials and methods section of the main paper. Three types of plasticware were tested, polystyrene petri dish (VWR, Radnor, PA, USA), Nuclon Delta petri dish (ThermoFisher) with surface treatment to adhere cells, and 50 ml low-binding tubes (Eppendorf SE, Hamburg, Germany). PBS and 100 µl of spray solution were added to each container and incubated for 10 min. An aliquot from each container was added to 10 ml NucliSENS lysis buffer and DNA was extracted and quantified as described in “Nucleic acid extraction and qPCR” in the materials and methods section in the main paper. The ratio between the concentration of each sample and control was used to identify if adhesion was an issue. A ratio below one would suggest that there was less PA after incubation compared to the control.

**Results**

The ratios between each sample and the control showed no clear signs of adhesion of PA. Nuclon petri dishes which were surface treated for maximum adhesion of cells had a ratio of 1.19, while 50 ml low-binding tubes had a ratio of 0.94 and polystyrene petri dishes 0.96.
